# Comprehensive care for people living with heart failure and chronic obstructive pulmonary disease—Integration of palliative care with disease-specific care: From guidelines to practice

**DOI:** 10.3389/fcvm.2022.895495

**Published:** 2022-09-27

**Authors:** Anna Kowalczys, Michał Bohdan, Alina Wilkowska, Iga Pawłowska, Leszek Pawłowski, Piotr Janowiak, Ewa Jassem, Małgorzata Lelonek, Marcin Gruchała, Piotr Sobański

**Affiliations:** ^1^1st Department of Cardiology, Medical University of Gdańsk, Gdańsk, Poland; ^2^Department of Psychiatry, Medical University of Gdańsk, Gdańsk, Pomeranian, Poland; ^3^Department of Pharmacology, Medical University of Gdańsk, Gdańsk, Pomeranian, Poland; ^4^Department of Palliative Medicine, Medical University of Gdańsk, Gdańsk, Pomeranian, Poland; ^5^Department of Pneumonology, Medical University of Gdańsk, Gdańsk, Pomeranian, Poland; ^6^Department of Noninvasive Cardiology, Medical University of Lodz, Łódź, Poland; ^7^Palliative Care Unit and Competence Centre, Department of Internal Medicine, Schwyz Hospital, Schwyz, Switzerland

**Keywords:** heart failure, chronic heart failure, chronic obstructive pulmonary disease, palliative care, advanced care planning

## Abstract

Heart failure (HF) and chronic obstructive pulmonary disease (COPD) are the leading global epidemiological, clinical, social, and economic burden. Due to similar risk factors and overlapping pathophysiological pathways, the coexistence of these two diseases is common. People with severe COPD and advanced chronic HF (CHF) develop similar symptoms that aggravate if evoking mechanisms overlap. The coexistence of COPD and CHF limits the quality of life (QoL) and worsens symptom burden and mortality, more than if only one of them is present. Both conditions progress despite optimal, guidelines directed treatment, frequently exacerbate, and have a similar or worse prognosis in comparison with many malignant diseases. Palliative care (PC) is effective in QoL improvement of people with CHF and COPD and may be a valuable addition to standard treatment. The current guidelines for the management of HF and COPD emphasize the importance of early integration of PC parallel to disease-modifying therapies in people with advanced forms of both conditions. The number of patients with HF and COPD requiring PC is high and will grow in future decades necessitating further attention to research and knowledge translation in this field of practice. Care pathways for people living with concomitant HF and COPD have not been published so far. It can be hypothesized that overlapping of symptoms and similarity in disease trajectories allow to draw a model of care which will address symptoms and problems caused by either condition.

## Introduction

Cardiovascular diseases (CVD) and respiratory diseases are the leading causes of morbidity and mortality in developed countries (1, 2). Among them, chronic heart failure (CHF) and chronic obstructive pulmonary disease (COPD) are regarded as the most common. Both diseases may cause serious clinical, social, and economic burden (1, 2). Heart failure (HF) affects up to 4% of the global population, and the number of new cases is constantly rising (1–3). The causes of this phenomenon are associated mostly with aging of the society and, paradoxically, with the improved survival in HF and acute coronary syndromes being one of common causes of HF (1). COPD is currently the third leading cause of death worldwide (2, 4). As smoking, which is a major risk factor for the development of COPD, as well as for coronary artery disease, is still widespread in the population, particularly of low and moderate incomes, an upward trend in the number of new cases of COPD is still predicted (2, 4). CHF and COPD having similar risk factors coexist in about 30% (range 9–52%) (2, 5, 6). If CHF and COPD coexist, the function of many organs and systems is affected more profoundly, the symptoms overlap, the quality of life (QoL) is limited more seriously, and the mortality risk is higher, particularly in the elderly (1, 2, 7). CHF increases mortality risk in people with COPD and CVD being the most common cause of death among this population, but the influence of COPD on all-cause mortality in the HF has not been proven so far (8, 9). The management of people living with CHF and COPD needs to address similar problems and symptoms and to prevent recurrence of exacerbations of these diseases (1, 5, 6). The guidelines recommend implementation of disease-specific management for HF and COPD even in the most advanced stages of disease. Interestingly, most of them recommend care provided by multidisciplinary team, including palliative care (PC), if needed (1–3, 10, 11).

Palliative care is an active, holistic approach that focuses on improvement of QoL of people living with life-threatening diseases that relieve health-related suffering (12, 13). Nowadays, it is dedicated to all suffering from a disease that does not fully respond to disease-specific management. It should be provided alongside the whole trajectory of living with the disease, regardless of diagnosis, prognosis, and risk of death (1–3, 10, 14–16). The core principle of PC is facilitating effective collaboration between the ill person, her or his family, and healthcare professionals from all disciplines and specialties involved in the care with the goal to empower the person living with a disease to achieve best possible QoL, according to personal values, to strengthen autonomy, and to secure maintaining dignity (17). PC acknowledges the relatives, often being informal caregivers, as subjects who need (PC) support, with the goal to maintain their QoL and facilitate their ability to care (11, 18). PC goes beyond alleviation of symptoms and perceives addressing psychological, spiritual, and social needs as integral aspects of care (1–3, 10, 14–16). Many scientific societies recommend that PC should be integrated in the care for people living with CHF and COPD as soon as the needs emerge. (1–3, 10, 14–16).

## Triggers for the implementation of palliative care for people living with advanced chronic heart failure and severe chronic obstructive pulmonary disease

The current guidelines recommend PC as a key component of multidisciplinary approach to people living with CHF and COPD, which should be applied based on needs, along with disease-specific management, regardless of the stage of these diseases and the expected survival (1–3, 11). Needs should be assessed as often as it is necessary, for example, in the case of significant changes in the course of the underlying disease, in general health, or in factors related to the person or his family, optimally using validates tools (1–3) (Table 1). The Needs Assessment Tool: Progressive Disease (NAT:PD) has been validated in cancer, HF, and interstitial lung disease and recently has been proposed as applicable in any progressive disease (19). It contains prompts to assess the needs and wellbeing of the ill person, the ability to care of relatives, and their wellbeing. The last prompt is dedicated to the consideration who will address existing needs (3, 20). Alternatively, other available validated scales assessing the presence and the severity of symptoms can be used to recognize indication for PC involvement (1–3). The most widely used tools are Numeric Rating Scale (NRS) and Edmonton Symptom Assessment Scale (ESAS) being in fact a list of NRS for nine most common and one self-determined symptoms, the Integrated Palliative Care Outcome Scale (IPOS) (3).

**TABLE 1:** The most important changes in the course of HF and COPD initiating the assessment of PC needs; based on: (1–3, 8, 11, 13, 132).

HF	COPD
• *De novo* HF with severe symptoms refractory to treatment • Symptoms of advanced CHF (NYHA IV) • Qualification for CIEDs • Qualification for TAVI • Eligibility for valve replacement surgery • Qualified for HTX or MCS	• *De novo* COPD with severe symptoms refractory to treatment • Symptoms of advanced COPD (category D, bronchial obturation GOLD 3/4, mMRC grade 3/4) • Eligibility for lung transplantation • Initiation of long-term oxygen therapy • Initiation of home non-invasive ventilation

• Progressive worsening of CHF and COPD with severe symptoms refractory to treatment • Care dependence and poor self-management • Frequent, recurrent exacerbations of CHF and/or COPD • Cachexia • Inability to attend cardiopulmonary rehabilitation • Survived cardiopulmonary resuscitation • Eligibility for heart and lung transplantation	

CIEDs, cardiovascular electrical devices; COPD, chronic heart failure; GOLD, global initiative for chronic obstructive lung disease; HF, heart failure; HTX, heart transplantation; MCS, mechanical cardiac support; MRC, medical research council; NYHA, New York heart association; TAVI, transcatheter aortic valve replacement.

**FIGURE 1:**
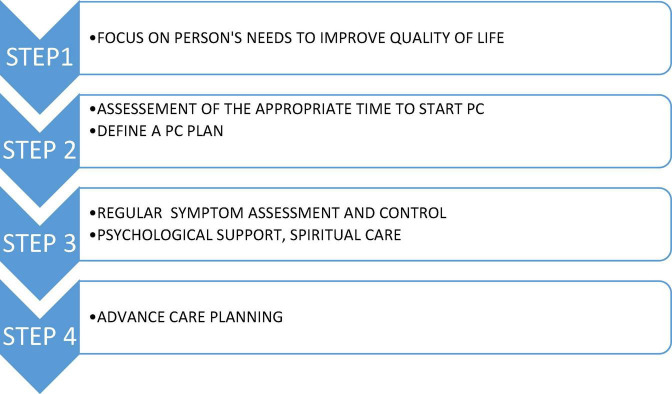
Steps in PC in people living with CHF and COPD; based on (1–3). CHF, chronic heart failure; COPD, chronic obstructive pulmonary disease.

The involvement of PC in care for people with heart and/or lung disease is less common than with cancer (18, 21, 22). The EPICTER study has shown that only 15% of patients hospitalized with advanced HF (23% of all HF hospitalizations) receive PC, but mostly only those with symptomatic cancer (as concomitant disease) in the last hours of life, after standard therapy has been exhausted (21, 23). The main causes for late PC involvement are depicted in Table 2 (3, 22–25). After the needs have been assessed, PC can be implemented stepwise. This process is presented below, with particular emphasis on the interdisciplinary approach in this group of patients (Figure 1).

**TABLE 2:** The most common causes of insufficient PC involvement in people living with COPD and CHF; based on (3, 16, 20–23).

Cause	Solution proposal
• Uncertain prognosis of COPD and CHF	• Collaboration in a multidisciplinary cardiopulmonary team to optimize standard care and choose the optimal time to start PC • Implementing of needs based model of triggering PC involvement
• Underestimation of the PC needs	• Regular assessment of the PC’s needs using available scales, both in primary and secondary care
• Person’s fear of talking about PC and end of life	• Improving awareness on PC principles in the society • Providing the patient with psychological, spiritual and social support in the scope adjusted to the needs based model of PC provision
• Insufficient physician’s communication skills on end-of-life related topics	• Training in clinical communication skills in non-PC specialists
• Incorrect perception of the PC as a solely care for the dying, lack of PC education	• Palliative care education and training programs for healthcare professionals
• Insufficient cooperation between PC specialists and other healthcare providers	• Meetings in a multidisciplinary group, including cardiologists, pneumonologists and PC specialists aimed at implementing an integrated PC

CHF, chronic heart failure; COPD, chronic obstructive pulmonary disease; PC, palliative care.

Although PC for those sub-populations exists, there is a lack of guidelines for the PC management of people living with COPD and coexisting CHF. This manuscript merges the available general and specific for either condition PC.

## Main clinical problems in people living with heart failure and chronic obstructive pulmonary disease

**FIGURE 2:**
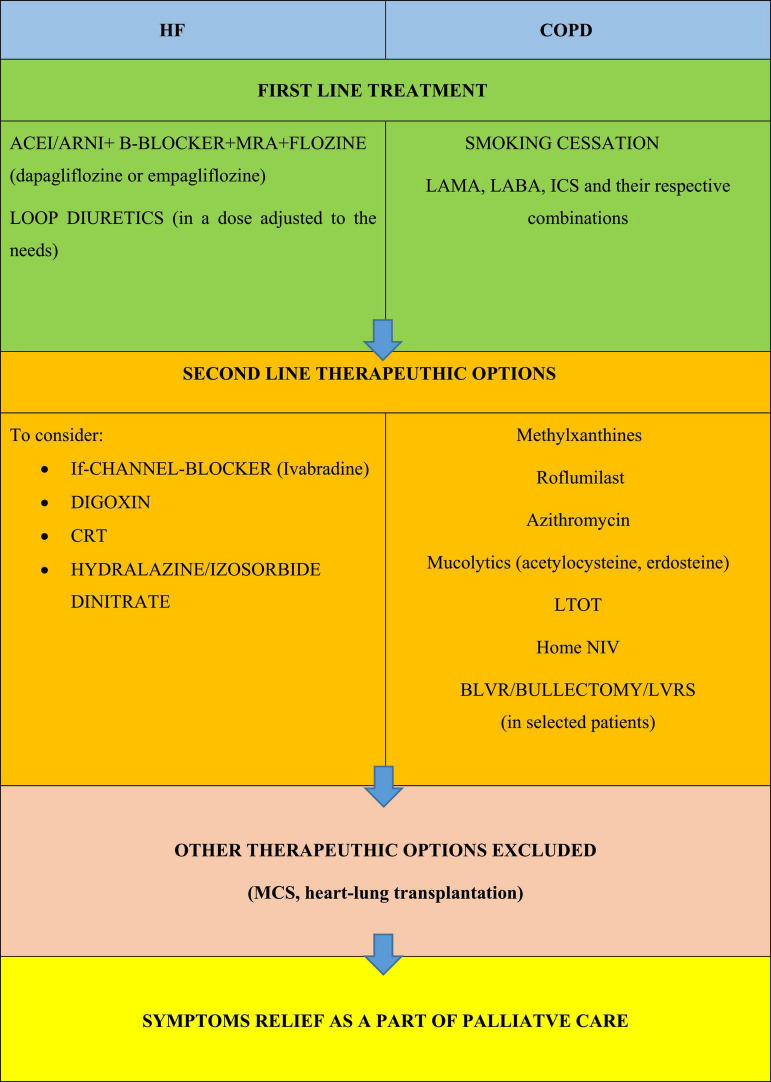
Principles of optimizing the treatment of the underlying disease in people living with COPD and HF; based on (1–3). ACE, angiotensin converting enzyme inhibitor; ARNI, angiotensin receptor neprilysin inhibitor; BLVR, bronchoscopic lung volume reduction; COPD, chronic obstructive pulmonary disease; CRT, cardiac resynchronisation therapy; CS, corticosteroids; HF, heart failure; ICS, inhaled glucocorticoids; LABA, long acting B_2_ agonist; LAMA, long acting muscarinic antagonist; LTOT, long term oxygen therapy; LVRS, lung volume reduction surgery; MCS, mechanical cardiac support; MRA, mineralocorticoid receptor antagonist; NIV, non-invasive ventilation; PDE-4, phosphodiesterase-4; SABA, short acting β agonist.

**FIGURE 3:**
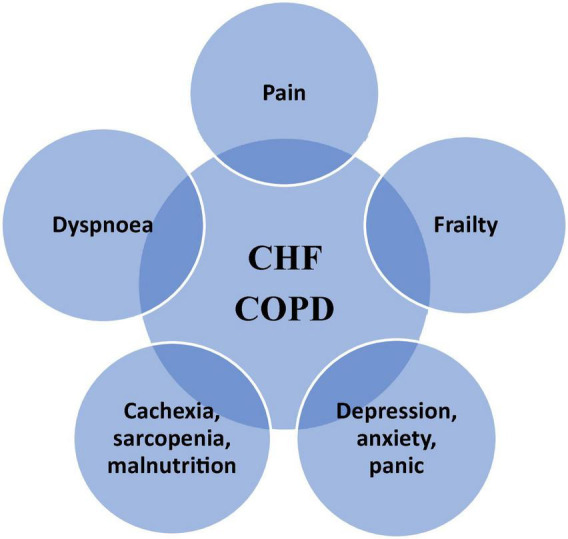
The most common clinical problems overlapping in CHD and COPD; adapted from (1–3). PC, palliative care.

The disease-specific management should be continuously optimized according to current guidelines, even in the most advanced stages of the disease and in people receiving care with the main focus on symptom relief. The applicability of this treatment and adjustment of treatment goals, according to given situation, is mandatory (1–3) (Figure 2). It should be emphasized that patients with COPD and CHF present many similar symptoms and experience many common problems that may overlap, constituting a therapeutic challenge for cardiologists and pneumologists (1–3, 5, 6) (Figure 3).

The symptomatic management of most common symptoms experienced by people with advanced CHF and severe COPD is discussed below.

### Breathlessness

**FIGURE 4:**
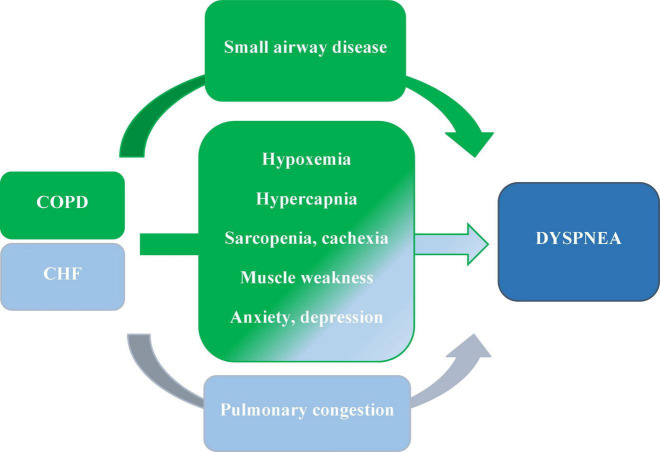
The major causes of dyspnea in people living with COPD and CHF coexistence; based on (1–3, 30).

Breathlessness is ubiquitously present in people living with CHF or COPD, beginning from very early stages of disease till the phase of dying. Disease-specific treatment alleviates it, but does not eliminate it completely, especially in advanced disease, when it is present all the time, despite optimal cardiological or pneumological management (1–3). The term “breathlessness” is commonly used to call the difficulties in breathing; however, people experiencing it use several descriptors to express their experience (e.g., tightness in the chest, air hunger, shortness of breath, shallow respiration, breathlessness, difficulty in breathing, heavy breathing, and feeling lack of air). It is regarded that this plurality of names describes the plurality of pathophysiological processes evoking breathlessness (1–3). Even if the factors causing breathlessness in heart and lung disease differ in less advanced stages, as the diseases progress, they increasingly overlap or even become common (1–3, 14, 26) (Figure 4).

The first step in alleviating breathlessness is optimization of treatment of the underlying disease or concomitant disease that can additionally aggravate it (like pneumonia, hydrothorax, or anemia). For many years, the intensification of β-blocker therapy in patients with HF and coexisting COPD has been controversial. Nevertheless, according to the current ESC and GOLD guidelines, these patients should be treated for HF in the same way as patients without COPD, including optimization of β-blocker treatment. Recent studies showed that β-blockers, particularly cardioselective ones, are safe in the treatment of COPD patients, reduce all-cause mortality, and might contribute to the reduction of COPD-related hospitalizations (27–29). On the contrary, long-acting anticholinergics and β_2_-agonists needed for COPD can evoke tachycardia, undesired in CHF. Optimization of treatment of each disease requires close monitoring of the function of the other one (30).

Improvement of the burden caused by breathlessness requires implementation of non-pharmacological management which may be especially successful if the breathlessness is of moderate severity (14, 26). In COPD, dyspnea exceeding point 2 on mMRC scale is an indication for general and pulmonary rehabilitation. In alleviation of chronic breathlessness training, the breathing technique adapted to the needs and possibilities of physical activity, cooling the face with a hand-hold fan directed at the triangle between mouth, nose, and cheeks, spraying a cold water on this area, psychological support, and education of patients and their relatives are considered as potentially helpful (1–3, 14, 26, 31). In COPD neuromuscular electrical stimulation and chest wall vibration, stimulation of mucus removal can be tried (2, 26). Oxygen can improve breathlessness in the majority of patients with COPD, sometimes even without documented hypoxemia, and in some patients with HF, predominately those with hypoxia (1–3).

The pharmacological, palliative treatment of chronic, refractory dyspnea is based on low doses of opioids (i.e., significantly lower, as used for pain management), mainly morphine. They have been studied predominantly in COPD and in cancer. Their efficacy and safety in HF are still not well-proven; however, they are recommended in cardiological guidelines (1–3, 14, 26, 32, 33). Morphine significantly alleviates breathlessness in about 50% of treated patients. The most commonly suggested initial dose is 10 mg/day p.o. that, if needed, can be up titrated gradually by 10 mg/day steps, every 7–10 days, to maximal 30 mg/day. Some experts recommend starting with much lower dose, what can be reasonable in elderly, cachectic, fragile, affected with comorbidities, with organ insufficiency (3, 34). The side effects, seen sometimes even with low doses of opioids, include constipation, usually transient nausea and vomiting, sedation/drowsiness, and addiction (1–3, 26). The most feared is respiratory depression that can be successfully prevented by proper dose titration and meticulous monitoring of symptoms and side effects as well as changes in clinical status and co-medication. The fear of opioid side effects (respiratory depression and addiction) together with lack of skills in their prescribing by non-palliative medical specialties is the most frequent cause of undertreatment of breathlessness (33). Benzodiazepines are commonly used for breathlessness alleviation, despite the lack of proven benefits and known harms of side effects. High doses can cause drowsiness and somnolence, especially in the elderly. The risk of addiction should not be neglected. Myorelaxant properties can impair respiratory muscle function and increase the risk of falls (1–3). Benzodiazepines may be considered as second- or third-line treatment of acute dyspnea in case of ineffectiveness of other therapeutic options, particularly in the presence of severe anxiety or in the dying (1–3). Mirtazapine is waking growing interest as a therapeutic option for people suffering for refractory breathlessness, especially those with concomitant anxiety, but randomized trials are underway (35).

### Pain

Pain is as common in people living with CHF and COPD as in people living with cancer, but its intensity is lower (36). That is why non-pharmacological management and non-opioids are more often sufficient, as in people with cancer. If the pain is severe, the addition of opioids should be considered. The non-steroidal anti-inflammatory drugs (NSAIDs) are contraindicated in people with HF, unless in very selected situation, the antiphlogistic effect is indispensable, as additionally to general sided effects (like gastrointestinal bleeding), they increase the risk of HF-related hospitalizations, renal function worsening, major atherothrombotic events, and death (1–3). The only exception among NSAIDs is acetylsalicylic acid (ASA), used at a dose of 75–100 mg/day as secondary prophylaxis in atherosclerosis (i.e., coronary artery disease). There are not known specific contraindications for their use in people with COPD, but administration of NSAIDs, including ASA, has been shown to increase risk of new atrial fibrillation or bleeding (37, 38). The randomized ENABLE-CHF-PC trial has shown that PC telehealth significantly improves pain intensity and its interference with daily life, but not QoL or mood (39).

### Sarcopenia and cachexia

Sarcopenia is a metabolic syndrome characterized by muscle loss, leading to diminished muscle strength, and performance, resulting in reduced mobility (40, 41). If sarcopenia is accompanied by unintended loss of more than 5% edema-free body weight within 12 months, cachexia should be diagnosed. Both conditions are seen in people living with advanced chronic diseases, with a prevalence 5–15% in advanced COPD and HF. The prevalence of sarcopenia, due to lack of universal clinical criteria, is more difficult to ascertain. Some authors suggest that it can affect 27% patients with COPD, with the prevalence growing with the advancement of the disease (42, 43). Among those living with HF, the prevalence of sarcopenia is 20% higher than in the age-matched healthy probands (44). It is associated with adverse outcomes, including falls, dysfunction, weakness, and death (42, 45). The most important factors leading to sarcopenia and cachexia are inadequate protein intake, malabsorption due to gut edema and hypoperfusion, metabolic imbalance, and physical inactivity (46, 47).

Nutritional screening should be performed in each patient with CHF and COPD using on of established tools: Nutritional Risk Screening 2002 (NRS 2002), Subjective Global Assessment (SGA), Mini Nutritional Assessment (MNA), Malnutrition Universal Screening Tool (MUST), Short Nutritional Assessment Questionnaire (SNAQ), for cachexia and SARC-F, SARC-calF test, and Mini Sarcopenia Risk Assessment (MSRA) for sarcopenia (48–55). Monitoring of weight is not a sensitive screening method as fluid retention can mask the loss of dry body weight and malnutrition. Useful for muscle function assessment is the handgrip strength measurement (56). Alternatively, body composition analysis (BIA), dual-energy X-ray absorptiometry (DXA), or muscle ultrasound may be used (47). BIA is a safe, reliable, inexpensive, and widely available tool (57). The main limitation of BIA and DXA in PC CHF/COPD patients is body water accumulation which may negatively affect the results (58).

Aerobic exercise and dietary interventions are suggested to prevent sarcopenia/cachexia, but there are no large studies in people with advanced CHF and COPD. The hypercaloric and hyperproteic supplementation of Dietary Approaches to Stop Hypertension (DASH) or Mediterranean diets are recommended, but this has not been evaluated in randomized studies (47). Participation in rehabilitation programs lead to improvements in exercise capacity and QoL (59).

### Exacerbations

Acute exacerbations of COPD (AECOPD) and acute decompensations of CHF (ADCHF) are the common cause of (re-)hospitalizations (1, 2). Nearly 20% of COPD patients and about 25% of CHF patients are re-hospitalized within 1 month (1, 2, 18, 24, 60). Each ADCHF and AECOPD are a potential life-threatening condition and significantly increase the risk of all-cause mortality and re-hospitalization (1, 2). Undoubtedly, the optimization of guidelines directed management for COPD and CHF is crucial for the prevention of disease exacerbations (1, 2). Preliminary reports suggest that PC provided as an element of holistic approach may also additionally contribute to reduction of the rate of exacerbation-related readmissions (21, 24, 61–65).

### Exercise rehabilitation

Exercise rehabilitation is an essential part of the multidisciplinary approach to people with HF and COPD recommended by ESC guidelines to all patients with HF and by GOLD experts to all patients with COPD who are able to undertake it (1, 2, 66). In population with HF, the rehabilitation improves physical capacity and QoL and reduces the frequency of HF-related hospitalizations (1, 2, 66–68). HF is a cause of only 15% of referral for cardiac rehabilitation, and as high proportion as 20% of patients with HF terminate the rehabilitation programs prematurely (69). Pulmonary rehabilitation, particularly involving aerobic exercises, increases physical capacity, reduces the feeling of dyspnea and fatigue, and significantly improves the QoL in people living with COPD (2, 14, 26, 70, 71). In patients with coexistence of CHF and COPD, exercise rehabilitation is especially needed, because in this group the decrease in cardiopulmonary capacity has a particularly negative impact on physical capacity, the severity of the dominant symptoms (particularly breathlessness), and the QoL (72, 73). The limitation of physical capacity cachexia and sarcopenia resulting in skeletal muscle dysfunction exaggerates breathlessness and fatigue in people with CHF and COPD (1, 2, 66). Exercise rehabilitation improves function of peripheral muscles, physical condition, symptoms of depression, and anxiety in people living with HF and COPD (14, 74, 75). Aerobic exercises or endurance training, inspiratory muscle training, or neuromuscular electrical stimulation (NMES) of lower limb muscles are especially recommended (76). The coexistence of COPD and CHF does not limit benefit of exercise rehabilitation (77). Exercise rehabilitation should be thus considered in all patients with HF and COPD, including severe forms of these diseases (1, 2, 66).

The rehabilitation can be part of an integrated PC in patients with COPD and CHF. Maddocks et al. and Reticker et al. suggest that PC and pulmonary rehabilitation have common goals (14, 78). The choice of the type, intensity, and duration of training should be tailored individually, with emphasis on the patient’s general condition and capabilities, the severity of the underlying disease, and the coexistence of comorbidities. Even in patients with severe HF and COPD, supervised exercise rehabilitation allows for individualization of training while maintaining a high level of safety (1, 2, 66). Telerehabilitation offers remote supervision of rehabilitation specialists (26, 79). Bernocchi et al. showed that with 4-month home telerehabilitation, the improvement with the distance in 6-min walk test (6MWT), the severity of dyspnea, the physical activity, and QoL can be achieved (80). Unfortunately, despite the clear benefits of simultaneous cardiac rehabilitation and pulmonary rehabilitation in COPD and CHF patients, implementation of cardiopulmonary rehabilitation in the form of integration of both programs is still challenging (81, 82).

### Psychiatric disorders

Diagnosing of depression in people living with advanced disease can be quite challenging, as anxiety, fatigue, loss of appetite, or insomnia can be caused by diminished physical capacity and mood disorder. Using Patient Health Questionnaire-9 (PHQ-9) can facilitate timely and proper recognition of this comorbidity (83).

Depression is common and affects up to 40% of people living with CHF and/or COPD (84, 85). Depression favors unhealthy living still, smoking, diminished activity, and weight gain (84). It exerts negative impact on the QoL and adherence to treatment and increases the risk of hospitalization and death, including suicides (85–88). For those reason, it is suggested to assess people affected by HF for depression and treat them if required (3). Adequate statements with COPD have not been published so far, but it can be hypothesized that the approach should be similar. The management should consist of non-pharmacological (cognitive behavioral therapy (CBT) and aerobic exercise) and pharmacological interventions (89, 90). Selective serotonin reuptake inhibitors (SSRI) and mirtazapine are considered the first choice (35). Sertraline and escitalopram have been shown to be safe in people with HF and sertraline gave promising results in COPD (91, 92). Citalopram can cause QTc prolongation, especially in higher doses and in older patients, that is why it should be prescribed with caution. SSRIs can cause hyponatremia in the mechanism of syndrome of inappropriate antidiuretic hormone secretion, especially when combined with thiazide diuretics. SSRIs co-administrated with ASA and/or clopidogrel increase the risk of bleeding. Fluvoxamine and fluoxetine increase the concertation of warfarin, but decrease the metabolism of clopidogrel to its active metabolite.

Bupropion, noradrenaline and dopamine reuptake inhibitor (NDRI), is an effective and safe antidepressant registered for smoking cessation, which is particularly significant in COPD patients. Importantly, bupropion is a substrate for CYP2D6 similarly to clopidogrel and can decrease digoxin level; therefore, serum concentration monitoring is required. Most SSRIs, duloxetine, and bupropion inhibit CYP2D6 causing increased exposure of beta-blockers, and their dose reduction might be required. Mirtazapine has an interaction of unknown mechanism with warfarin causing increased risk of bleeding (93). Mirtazapine can also cause somnolence and weight gain which in some cases, however, can be beneficial. Medications used in COPD like beta-agonists used with SSRIs, mirtazapine, and trazodone can induce QT prolongation. Muscarinic antagonists can increase the risk of delirium and symptoms like dry mouth, constipation, and urinary retention which can be also caused by mirtazapine and trazodone (94). SSRI and NDRI—like venlafaxine and duloxetine—can cause hypertension and prolong QT interval. Tricyclic antidepressants and monoamine oxidase inhibitors are contraindicated in HF due to effect on blood pressure and QT prolongation, trazodone although trazodone is useful as a hypnotic agent not causing addiction should not be used in patients with ventricular arrhythmias (84, 93). A good strategy is to “start low, go slow” considering decreased drug metabolism. A new antidepressant strategy is ketamine—NMDA antagonist which turned out to be effective, rapid-acting add-on treatment in resistant depression (TRD). Its intranasal enantiomer es-ketamine has been recently approved by the FDA as an add-on treatment in TRD. Cardiac safety of add-on es-ketamine was evaluated in 1700 patients with TRD (95). Other experimental treatment used in depression and existential distress in patients with life-threatening disease is psilocybin-assisted psychotherapy. An RCT found reduction in the level of depression, suicidal ideation, and hopelessness after single dose of 0.3 mg/kg psilocybin administered in conjunction with psychotherapy (96). The results of recent Omega-3 Supplementation for Co-Morbid Depression and Heart Failure Treatment (OCEAN) trial revealed that high dose omega-3 was associated with improvement in cognitive depressive symptoms, social functioning, and 6MWT in depressed patients with HF (97).

### Anxiety

General anxiety disorder and panic disorder are common, especially in COPD patients and in patients with implantable cardioverter defibrillator (ICD) (85, 98). The Hospital Anxiety and Depression Scale (HADS) is a good tool to screen hospitalized patients for anxiety and depression (99, 100). In COPD patients, CBT is recommended as an effective non-pharmacological treatment for anxiety (101). Pharmacotherapy, apart from SSRIs, which are the first-line treatment for anxiety disorders, includes also buspirone (93). BDZ is generally not recommended in elderly and patients with HF due to risk of falls and delirium (102, 103). Alprazolam—short acting benzodiazepine, has an interaction with amiodarone which inhibits its metabolism through CYP3A4 and can cause enhanced alprazolam effects (93).

Insomnia is common and requires efforts to remediate the underlying cause if it is possible. When pharmacotherapy is needed, antidepressants and melatonin can be considered.

### Delirium

Delirium is an acute neuro-psychiatric condition caused by global brain dysfunction reaching prevalence up to 40% in palliative setting. The hallmark of delirium is disturbance in attention and awareness (104). Typically, delirium has acute onset and fluctuating course. Delirium is often underdiagnosed or misdiagnosed. A useful screening tool recommended by National Institute for Health and Care Excellence (NICE) is Confusion Assessment Method (CAM) (105, 106). Etiology of delirium is multifactorial, but most common triggering factors are infection, substance withdrawal, electrolyte disturbance, hypoxia, dehydration, anemia, hypo/hyperglycemia, organ dysfunctions, neurological diseases, and medications. Delirium correlates with increased mortality, morbidity, longer hospitalization, and higher costs of treatment. It increases the risk of falls and significantly disturbs the process of communication with the person (107). According to NICE guideline, prevention strategies like avoiding unnecessary catheterization, optimizing sleep conditions, encouraging physical activity, avoiding sensory deprivation (glasses, hearing aids), and using clock and calendar for orientation can be very effective (105). There are data on melatonin as an effective and safe preventing therapy in older patients undergoing surgical procedures (108).

The main goal in treatment should be identifying and cessation of the source of delirium. About 50% cases of delirium in palliative patients can be reversed with good communication at the end of person’s life (107, 109). No medication is registered for delirium treatment, but if the person is distressed or considered risk to themselves or others, low dose and short-term haloperidol or olanzapine can be considered (105, 110).

### Spiritual care

Spirituality is the dynamic dimension of human life that relates to the way persons experience, express and/or seek meaning, purpose, and transcendence, and the way they connect to the moment, to self, to others, to nature, to the significant, and/or the sacred (111).

Living with progressive disease confronts people with spiritual issues, that is, why spiritual care is integral part of PC. It supports people in coping with existential questions. As the spirituality, especially in West-European countries goes beyond religiously, spiritual care needs to be provided by, in addition to chaplains and pastoral care workers, all healthcare professionals offering their therapeutic presence. This kind of care is based on relations between the patient, caring team, and patients’ close ones (3). Evidence shows that spiritual counseling improves QoL in patients with HF (112). Spiritual peace along with healthy lifestyle has been shown to predict 5-year mortality better than functional status and comorbidity in people with HF (113).

### Advance care planning

Advance care planning (ACP) is a process of preparedness for the decision-making for the future, whereby individuals identify their goals and preferences concerning future care and treatment as well as discuss these goals and preferences with healthcare providers and family (114). It is aimed to ensure medical care the person receives, especially at the end of life, aligns with their preferences (115). During ACP process, the person is invited to reflect her or his personal values and goals, and based on this, to try to foresee what from applicable treatment and care options could be concordant with those values and goals in future, usually in the hypothetical end-of-life situation (116). Open and honest clinical communication helps to get realistic insight in current disease-related situation, the risk of progression, and chances for improvement in case of treatment success but as well for deterioration in case it fails (117, 118). The ACP decisions should be recorded and revised or update if appropriate (119). The outcome of the ACP process can be just being prepared for the moment of decision-making, writing advance directives (AD), sharing the conclusions with family (i.e., advance statement) without preparing any formal document, or asking the treating physician to prepare orders for life-sustaining therapies (Physician Orders for Life-Sustaining Therapies, POLST) (120). The AD can summarize the treatment or care options expected or unwished and/or indicate formal representative who will make medical decisions (surrogate) for the case the person loses decision-making capacity. The appropriate form of expression own will in given country is matter of local law and local traditions (121–123).

The ACP process in people living with HF and COPD should, additionally to general topics like hospital admission, or tube feeding, address disease-specific issues like ventilation, modification of cardiovascular implantable electronic devices (CIEDs), and/or mechanical circulatory support (124). The decision how to proceed with ventricular assist device (VAD) in case of serious advance events like refractory sepsis, cerebral bleeding, or cerebrovascular embolic insult is especially challenging, as most of affected people lose their decision-making capacity in such situations (125).

People with cardiovascular and pulmonary diseases prefer the early initiation of ACP conversations, that is, at the time of diagnosis and at transition points in the follow-up (126, 127). In practice, healthcare providers often initiate ACP process at the advanced stage of illness only (128, 129).

### Composition of palliative care team

For the adequate provision of specialized PC services, a qualified multidisciplinary team is required (130). The core team consists of physicians and nurses with specialized training, whereas psychologists, physiotherapists, social workers, and spiritual care workers constitute the extended, multiprofessional PC team. Other important contributors include psychologists, office workers, bereavement counselors, wound management and lymphedema specialists, occupational, art and speech therapists, dietitians, pharmacists, complementary therapists, trainers, and librarians (131). To ensure cohesive continuity of care, all involved including the person living with a disease and her or his family need to create a network (130, 132). Trained volunteers are important members of the therapeutic team, supporting services provided by professionals (133). Close cooperation of cardiology and/or pulmonology team, certified PC nurse, and physician assure achievement of better QoL and symptom relieve in people living with HF and COPD (134, 135).

### Organization of palliative care

Every person with needs has a right to get access to PC and pain as well as other symptom relief. To realize this, governments should facilitate integration of PC in healthcare systems and healthcare insurances need to reimburse the service (136, 137). PC can be provided on two levels: PC approach (called as well generic PC) and specialist PC (13). In case of PC approach, all healthcare professionals used to be engaged in the management of the person living with a disease implement basic PC principles in the care they usually provide, in a place the person has been cared for so far (e.g., general hospitals, cardiology or pulmonology units, outpatients clinics, and nursing homes). The specialist PC is provided by healthcare professionals having special training, provision of PC is their main business, this level of care should be provided to people with complex needs, or PC approach has appeared as not enough sufficient. Specialist PC should optimally be served by multiprofessional team, but acceptable can be engagement of physician and nurse with PC expertise cooperating with such a team (138). In many countries, there are also hospital PC support teams, providing an advisory service to hospital staff (139).

## Modification of cardiovascular implantable electronic devices activity at the end of life

Growing number of people living with CHF, also those with coexisting COPD, had received one of CIEDs, which, when the end-of-life approaches, can require a special attention to prevent device related suffering and/or providing futile therapies. The family of CIEDs includes antibradycardia pacemakers, cardiac resynchronization therapy (CRT) pacemakers, ICDs, or combination of them (CRT + ICD = CRT-D). They are originally implanted to improve the QoL (antibradycardia pacing and CRT) and/or prevent sudden cardiac death (ICD and CRT-D). Pursuing the main goal of healthcare—assuring the best possible QoL, during the whole life, even its last phase–dying, requires adjustment of all ongoing therapies, including the electrotherapies provided by CIEDs. Modification of CIEDs activities is aimed to prevent unneeded suffering caused by under- or over-treatment. Keeping active the whole devices or their functions that prevent, in unnoticeable for the patient way, bradycardia- or pauses-related symptoms or improve the synchrony of heart contraction, has from a medical perspective never to be questioned. On the contrary, the high voltage antitachycardia therapies (cardioversion/defibrillation), usually painful, and in the dying phase (when the death is not consequence of tachyarrhythmia) futile, should be considered as medically not indicated and discontinued (after receiving patient’s consent). Low-voltage antitachycardia pacing, even if adequate and unnoticeable, can prolog the dying, as terminating of tachycardia, even if potentially lethal, cannot save the life of person dying for otherwise end-stage disease (not for arrhythmia). When impending of the death can be anticipated, the sense of device activity must be evaluated and discussed with affected person. If the death cannot be avoided, and the potential arrhythmia is not the cause, but the mode of deaths, terminating it should be considered as prolonging dying, and not saving life (140–142). The deactivation of shocking function should be performed at the end of life, but the communication on this should happen much earlier, optimally even before implantation of CIED. Decision on modification of CIED activity should be integral part of advance care planning (141, 143). Communicating this is regarded by many clinicians as more challenging than withdrawal from other life-sustaining therapies (144). Clinicians should be educated that ICD deactivation is not a form of euthanasia and their decision should be supported by healthcare provider organizations with policies of management of devices with an option of planned device inactivation (140).

## Conclusion

Chronic heart failure and COPD are common, often coexist, and cause similar symptoms that cannot be completely alleviated with optimal guidelines driven disease-specific treatment, especially in advanced stages. The persistent symptoms caused by either condition limit the QoL and are a cause of suffering that can potentially be addressed by PC. The PC should be understood as additional layer of care, provided additionally to the disease-specific treatment, but not its alternative, that should be applied always when the needs emerge, independent of expected length of life/risk of dying. In people affected by HF and COPD, the close cooperation between cardiologists, pneumonologists, palliative medicine specialists, general practitioners, and other specialists, if necessary, is crucial. If the needs are complex or have not been efficiently addressed by multidisciplinary team, the involvement of specialized PC team should be considered.

## Author contributions

AK, MB, PS, AW, IP, LP, PJ, and EJ wrote sections of the manuscript. All authors contributed to conception and design of the study, manuscript revision, read, and approved the submitted version.
